# Prognostic DNA methylation markers for hormone receptor breast cancer: a systematic review

**DOI:** 10.1186/s13058-020-1250-9

**Published:** 2020-01-31

**Authors:** Tim C. de Ruijter, Frank van der Heide, Kim M. Smits, Maureen J. Aarts, Manon van Engeland, Vivianne C. G. Heijnen

**Affiliations:** 1 Division of Medical Oncology, Maastricht University Medical Center, PO Box 5800, 6202 AZ Maastricht, The Netherlands; 2 GROW – School for Oncology and Developmental Biology, Maastricht University Medical Center, 6200 MD Maastricht, The Netherlands; 3 Department of Pathology, Maastricht University Medical Centre, 6202 AZ Maastricht, The Netherlands

**Keywords:** Biomarkers, DNA methylation, Breast cancer, Hormone receptor positive, Oestrogen receptor positive, Luminal breast cancer, Prognosis, Promoter CpG island methylation, Survival

## Abstract

**Background:**

In patients with hormone receptor-positive breast cancer, differentiating between patients with a low and a high risk of recurrence is an ongoing challenge. In current practice, prognostic clinical parameters are used for risk prediction. DNA methylation markers have been proven to be of additional prognostic value in several cancer types. Numerous prognostic DNA methylation markers for breast cancer have been published in the literature. However, to date, none of these markers are used in clinical practice.

**Methods:**

We conducted a systematic review of PubMed and EMBASE to assess the number and level of evidence of published DNA methylation markers for hormone receptor-positive breast cancer. To obtain an overview of the reporting quality of the included studies, all were scored according to the REMARK criteria that were established as reporting guidelines for prognostic biomarker studies.

**Results:**

A total of 74 studies were identified reporting on 87 different DNA methylation markers. Assessment of the REMARK criteria showed variation in reporting quality of the studies. Eighteen single markers and one marker panel were studied in multiple independent populations. Hypermethylation of the markers *RASSF1*, *BRCA*, *PITX2*, *CDH1*, *RARB*, *PCDH10* and *PGR*, and the marker panel *GSTP1*, *RASSF1* and *RARB* showed a statistically significant correlation with poor disease outcome that was confirmed in at least one other, independent study.

**Conclusion:**

This systematic review provides an overview on published prognostic DNA methylation markers for hormone receptor-positive breast cancer and identifies eight markers that have been independently validated. Analysis of the reporting quality of included studies suggests that future research on this topic would benefit from standardised reporting guidelines.

## Introduction

In women, breast cancer is the most frequently diagnosed cancer worldwide, with an incidence of 1.7 million cases each year [1]. Most cases, 75–80%, are hormone receptor positive, meaning that tumour cells express the oestrogen receptor (ER) and/or the progesterone receptor (PR). Curatively treated breast cancer patients are at risk of recurrence of disease. This occurs in approximately 10% of patients with hormone receptor-positive breast cancer within 5 years and continues to be a risk with an annual rate of 1.4–2.2% over more than 20 years [2, 3]. Adjuvant systemic treatment diminishes the risk of recurrence, but can have adverse effects that negatively impact quality of life [4].

The risk of recurrence in current clinical practice is estimated by considering classical prognostic factors, using nomograms such as the UK-based PREDICT tool or New Adjuvant Online [5–7]. Despite the success of these risk prediction models to identify patients at high risk of recurrence based on clinical characteristics, prediction is on a population level and as a result leads to over- and undertreatment at a patient level [8]. Prognostic biomarkers may improve the risk assessment, making it possible to better distinguish patients with a high risk of recurrence who may benefit from additional treatment, from patients with a low risk of recurrence for whom additional treatment may be omitted [9]. This principle was recently demonstrated for both the Mammaprint and Oncotype DX biomarker assays by the MINDACT and TAILOR trials [10, 11].

Biomarker research has increasingly incorporated epigenetic processes, particularly DNA methylation. DNA methylation is the addition of a methyl group to the carbon 5-position of cytosine within a cytosine guanine (CpG) dinucleotide. As methylation is a common and early event in cancer, and DNA methylation patterns differ between breast cancer molecular subtypes [12, 13], alterations in the methylome form a potential class of biomarkers for early detection, prognosis and prediction to therapy [14–16].

At the moment, DNA methylation markers are not yet being used in the clinical setting of breast cancer, despite the fact that many studies focused on the potential prognostic role of these markers and many DNA methylation markers have been suggested to have prognostic value [17, 18]. Currently, an overview of these studies describing potential prognostic markers is lacking. In this systematic review, we provide a comprehensive overview of potential prognostic DNA methylation biomarkers for hormone-sensitive breast cancer. In addition, we comment on various methodological aspects of these biomarker studies, aiming to provide guidelines for optimising research into this subject.

## Methods

### Review format

This systematic review was conducted according to the Preferred Reporting Items for Systematic Reviews and Meta-Analyses (PRISMA) statement [19]. No review protocol was previously published.

### Eligibility criteria and study selection

Eligible articles were original research reports in the English language that had investigated hypo- or hypermethylated biomarkers in relation to patient survival or surrogate endpoints such as disease-free survival in breast cancer populations with oestrogen and/or progesterone receptor-positive breast cancer cases. We excluded in vitro studies, studies on non-human material, studies that focused on hereditary breast cancer cases, studies that focused on non-CpG DNA methylation and studies that had reported large amounts of data from biomarker arrays without further specification of the data to a single potential biomarker or biomarker panel.

### Search strategy

PubMed and EMBASE were searched up to November 2018 for eligible studies using the following keywords and equivalents of these: ‘breast cancer’, ‘DNA based methylation biomarker’, ‘hormone receptor positive’ and ‘prognostic or predictive clinical outcome’ (see Additional file 1: Table S1 for a complete overview of the search terms).

Two reviewers (FH and TR) independently selected studies based on title, abstract and in selected cases full text. Disagreement was resolved by discussion between the reviewers until consensus was reached. References of selected studies were crosschecked for additional studies that were eligible for inclusion.

### Data collection and extraction

The following data from all selected studies was collected independently by two reviewers (FH and TR): year of publication, study design, study population, length of follow-up, assay type and cut-off used, sequence of primer or probe, statistical methods used and reported association between marker and patient outcome were collected from all selected studies. When available, both univariate and multivariate outcome measures were collected. Study population information consisted of population size, country of patient selection, age, grade, hormone receptor status, HER2 status and stage according to the reported American Joint Committee on Cancer classification [20]. The level of evidence (LOE) was assessed for each publication according to criteria as defined by Hayes et al. [21] and the OCEBM Levels of Evidence Working Group [22].

For each publication, all study endpoints on outcome were collected and compared with ‘the proposed Standardized definitions for Efficacy EndPoints in adjuvant breast cancer trials’ (STEEP) [23]. Endpoints not defined in accordance to STEEP definitions were converted to STEEP-defined endpoints when sufficient information was provided. All defined biomarkers were checked for aliases in the NCBI Gene database and were reported by their current RefSeq gene names.

### Analysis of reporting

All selected articles were scored according to the ‘REporting recommendations for tumour MARKer prognostic studies’ (REMARK) criteria [24, 25]. The REMARK checklist consists of 20 items containing one or multiple sub-items. A single item was scored with one point if all relevant sub-items were reported, half a point if only part of the information was reported or zero points if no information on this item was reported. The REMARK checklist is presented in Additional file 2: Table S2. Scoring was performed by two independent researchers (FH and TR). If the total score per article differed, the differences were discussed until agreement was achieved.

REMARK scores were used to assess the risk of potential selection, measurement and confounding bias. The risk for selection bias was assessed by REMARK item #2 (‘patient characteristics’) and #6 (‘sample selection and follow-up’). Studies obtaining < 1.5 points for these combined items were considered to have an increased risk. Measurement bias regarding the assay method was assessed using REMARK items #5 (‘assay method’) and #11 (‘handling of marker values’). REMARK item #7 (‘clinical endpoint definition’) was employed to assess the risk of measurement bias regarding outcome assessment; incomplete or lack of reporting of this item (score < 1) was considered at risk for measurement bias. Confounding bias was assessed using REMARK criterion #16 (‘multivariable analysis’), as in multivariate analysis (score = 1) potential confounding is taken into account. In order to investigate the effect of study design on marker significance, we compared REMARK scores between studies that found significant results and studies that did not find significant results using a Wilcoxon signed-rank test.

### Forest plots

A forest plot was prepared for all methylation markers that were investigated in two or more study populations. When included studies reported results for more than one location per marker or reported results derived from more than one source of DNA, such as primary tumour tissue or blood serum, all reported results were represented in the forest plot. If available, multivariable hazard ratios (HRs), 95% confidence intervals (CI) and *p* values were used. When studies reported only *p* values without HRs, these were still included in the forest plot, in order to give a complete overview. The statistical programming language R (version 3.3.1) was used to perform all analyses and generate the figures.

## Results

### Search results

**Fig. 1:**
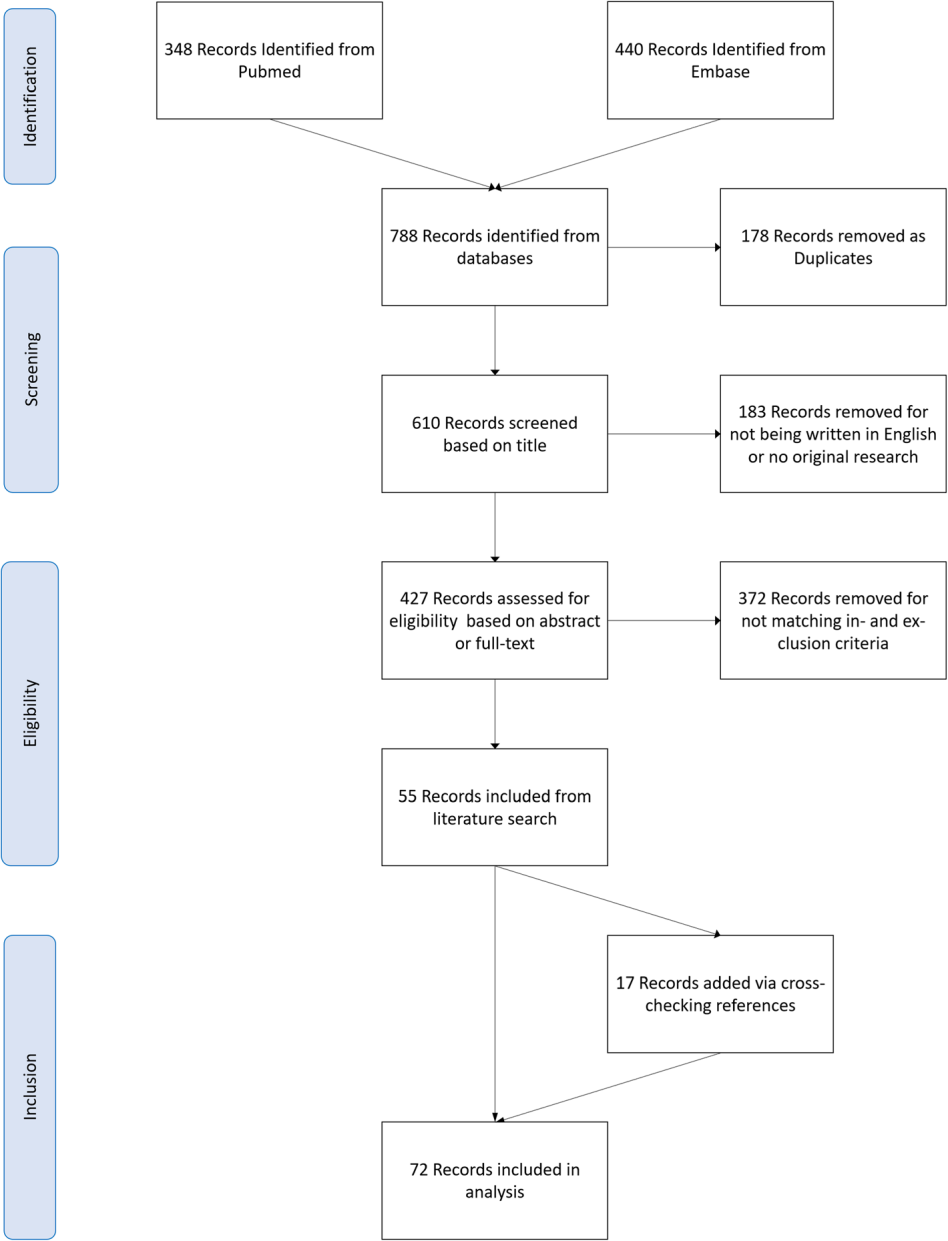
Flowchart showing the study identification process. In total, 72 studies were included in this systematic review

The search in the PubMed and EMBASE databases yielded a total of 788 potential publications. One hundred seventy-eight publications were removed as duplicates. After removal of 183 publications that either were not written in English or did not concern original research, 427 studies remained and were screened for eligibility based on title, abstract or full text. Three hundred seventy-two papers were excluded for not matching our inclusion and exclusion criteria. In addition to the remaining 55 papers, 17 papers were identified during reading and included in this review. This selection procedure resulted in 72 included papers [13, 20, 26–95]. A flowchart of this selection procedure is provided in Fig. 1.

### Study characteristics

A summary of all studies, studied markers and study characteristics is provided in Additional file 3: Table S3. The number of included patients ranged between 34 and 1163, with a median study sample size of 119. Median or mean follow-up time per study ranged between 20 and 238 months. In 59 studies (82%), either fresh frozen or formalin-fixed paraffin-embedded primary tumour tissue from surgical resections was used for DNA extraction. Nine studies (13%) used plasma or serum derived from the blood and one study (1%) used serum derived from the bone marrow. Fine needle aspirates of the tumour were used in one study (1%). Two studies (3%) did not report the origin of the DNA samples. None of the studies reported selection of a specific tumour grade or stage. All but three papers studied hypermethylation as opposed to hypomethylation. Ten different techniques were applied to assess methylation status, of which methylation-specific PCR was used most frequently (*n* = 33, 46%) followed by quantitative methylation-specific PCR (*n* = 16, 22%).

We could categorise 55 papers as OCEBM level 4 and 19 studies OCEBM level 3. There were no level 1 or 2 studies.

### Quality assessment

All included publications were scored for adherence to the REMARK criteria. Scores ranged from 4.5 to 19 out of the maximum 20 points; the median REMARK score was 12. Only four studies (6%) scored over 15 points.

**Fig. 2:**
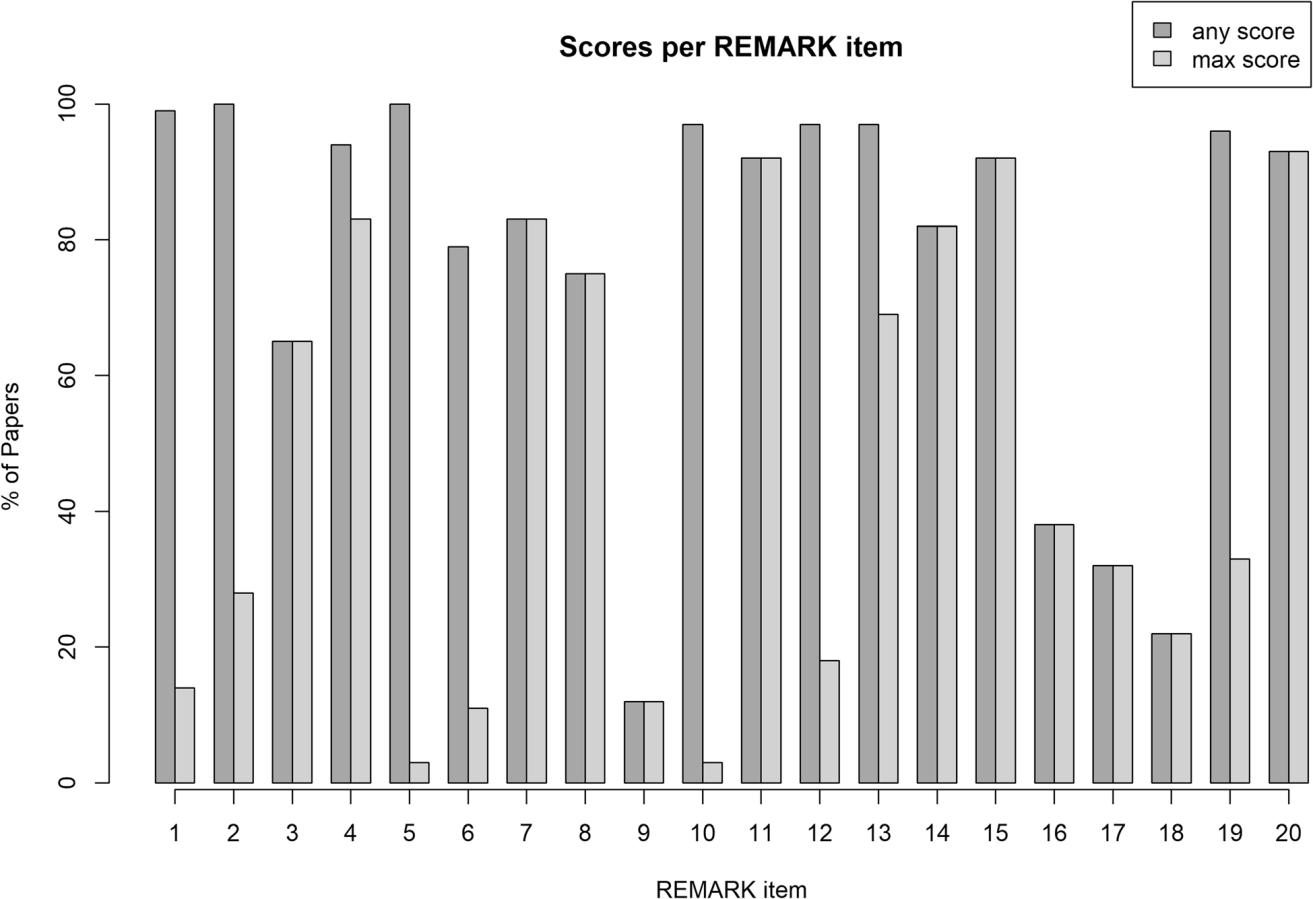
Quality assessment of included studies. The histogram depicts the completeness of reporting per remark item. The percentage of studies that reported any information is reported per item, as well as the percentage of studies that reported all required information

Quality of reporting highly differed per REMARK item. Figure 2 depicts the number of papers that provided any information for each REMARK item and the number of papers that provided all information per item as required by the REMARK guidelines. For most items, information was provided by the authors, but often this information was incomplete. For example, all studies provided information on the study population, but in 72% of the papers, clear inclusion and exclusion criteria or key patient characteristics, needed to place study results into a clinical perspective, were lacking. The assay used to analyse DNA methylation was mentioned in all studies; however, only two studies (3%) provided all information required to reproduce the assay. Similarly, all studies provided information on statistical analysis, but information needed to reproduce the analyses such as handling of missing data and selection of variables was missing in over 95% of the included papers. Other items, such as patient treatment (65%), biological origin of samples (83%), handling of cut-off values (92%) and demographics of included patients (75%), were generally well reported. Correlations between markers and classical prognostic factors, as well as univariate outcome results were adequately provided by most authors. However, multivariable analyses adjusting for classical prognostic markers were performed in only 32% of included papers.

Comparison of REMARK scores between studies that produced significant results and studies that did not produce significant results showed that studies with low REMARK scores were less likely to report a statistically significant correlation between the investigated marker and disease outcome (*p* = 0.007). The risk of bias of each included study is summarised in Additional file 4: Table S4.

### Prognostic marker findings

**Fig. 3:**
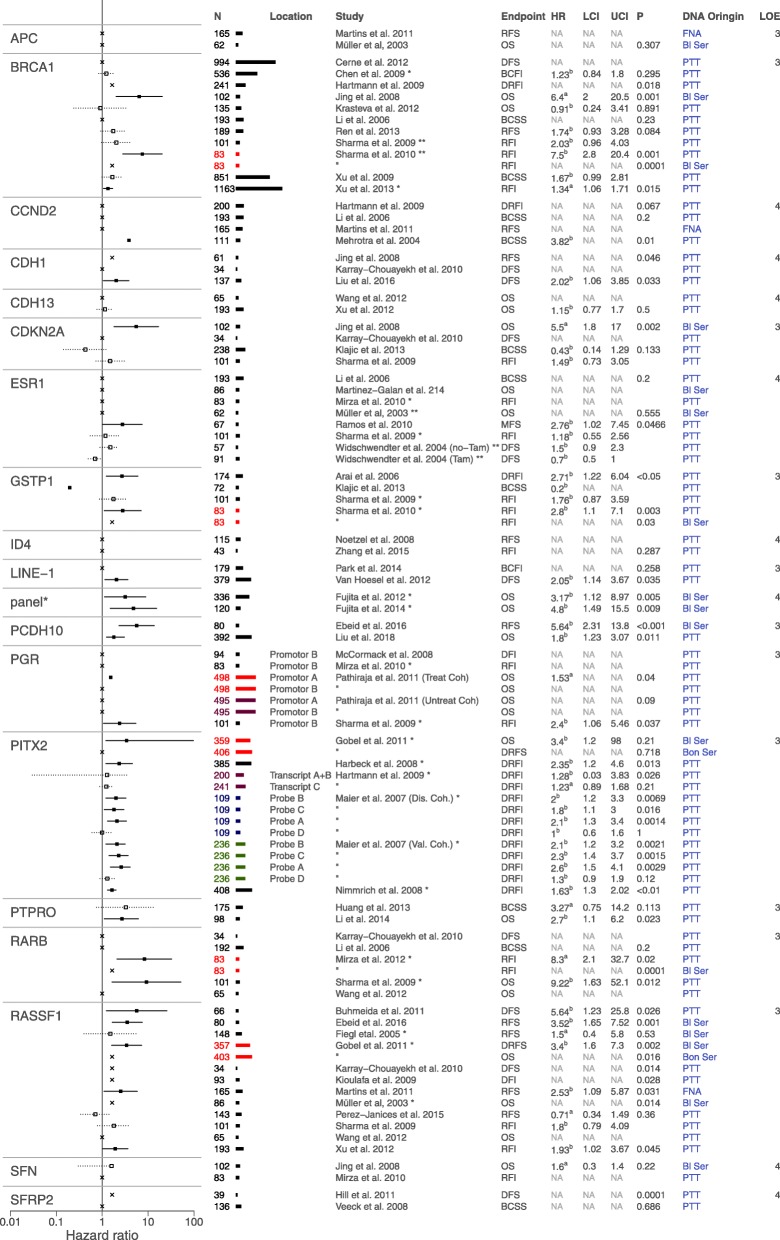
Forest plots of all methylation markers reported in two or more independent study populations. HRs with a statistically significant association are depicted with a solid line; HRs of reported markers with no significant association are depicted with a dotted line; univariate HRs (^a^) and confidence intervals (CI) are reported unless multivariate HRs (^b^) were available. Per marker, if results are derived from the same cohort, but with differing characteristics, such as differing DNA origin or location of methylation, this is represented by a coloured population bar. Per marker, if results originated from the same research group, this is indicated by an asterisk (*). Due to the vast number of individual results for these markers, for visualisation purposes, per marker, this figure shows one result per investigated population and tissue type. For a full representation of markers reported in two or more independent study populations, see Additional file 5: Table S5

**Fig. 4:**
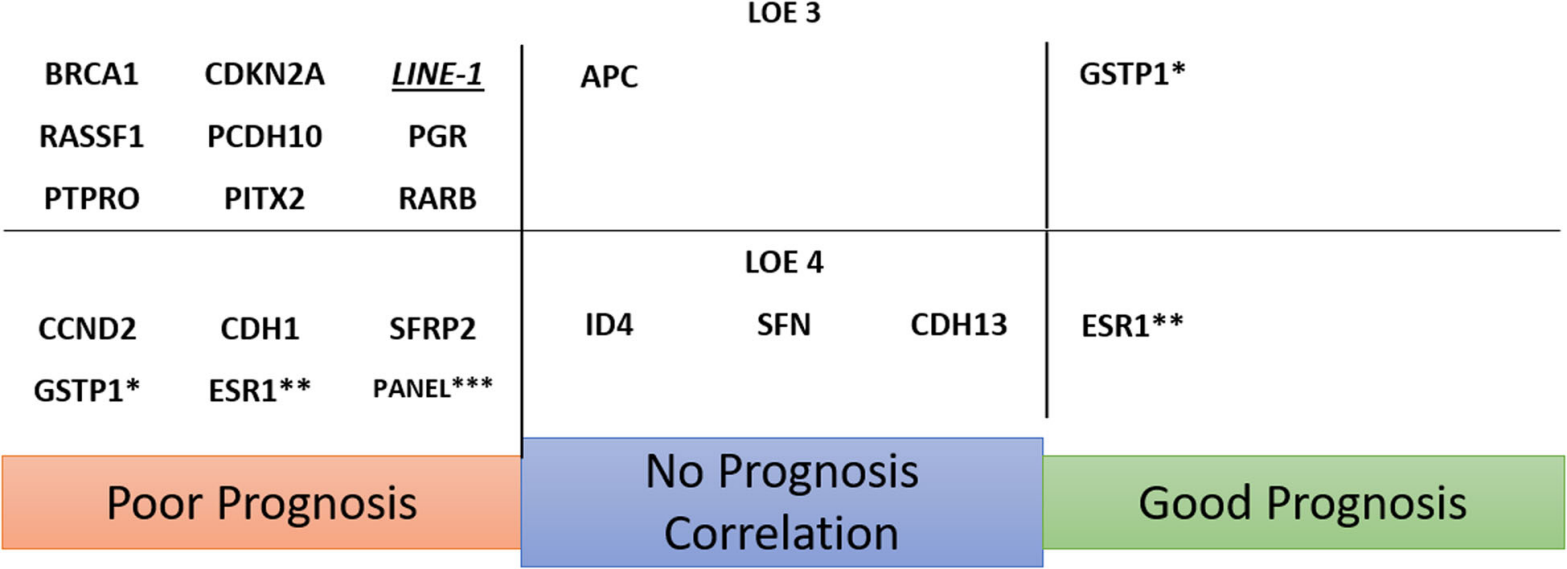
Methylation markers studied in at least two independent populations, separated by relation to prognosis and achieved LOE. Underlined markers were analysed as hypomethylation markers. Italic markers do not correspond to Ref-Seq registered genes. Markers GSTP1 (*) and ESR (**) were both significantly correlated with good and poor prognosis in separate studies. The mentioned panel (***) is a multigene panel consisting of markers GSTP1, RASSF1 and RARB

The 72 included studies reported on 87 different DNA methylation markers. Of these, 18 single markers were studied in more than one independent population. Forest plots summarising the results of these repeatedly studied markers are depicted in Fig. 3 and Additional file 5: Table S5. Hypermethylation of seven markers and one marker panel consisting of three markers was independently significantly associated with poor disease outcome [28, 32, 38–41, 48, 50, 52, 58, 61, 63, 66–69, 78, 79, 88, 92, 94]. Two markers were both significantly associated with poor and improved outcome in separate studies [54, 75, 78, 91]. Five markers showed a significant relation to poor outcome in one study, while other studies looking into the same marker found no correlation [44, 57, 58, 65, 83]. Finally, four markers showed no significant relation to disease outcome in any study [48, 63, 66, 68, 70, 88, 92, 95]. An overview of all markers studied in two or more independent populations is presented in Fig. 4. Of the 87 reported markers, 68 were only studied once in a single population. An overview of these markers is provided in Additional file 6: Figure S6 and Additional file 7: Table S7.

Hypermethylation of the markers *RASSF1*, *BRCA*, *PITX2*, *RARB*, *PGR*, *CDH1* and *PCDH10*, and the marker panel consisting of markers *GSTP1*, *RASSF1* and *RARB* showed a statistically significant correlation with poor disease outcome. *RASSF1* is the most extensively studied breast cancer methylation marker and was studied in 12 independent study populations [28, 32, 36, 40, 50, 52, 63, 68, 74, 79, 88, 92]. Eight studies found statistically significant results; all showed *RASSF1* methylation to be associated with poor survival (HR ranging from 1.93 to 5.64). The remaining four studies found no statistically significant association. *RASSF1* methylation was tested in DNA derived from primary surgical resections, blood serum, bone marrow-derived serum and in fine needle aspirates, and was capable of predicting outcome independently of DNA origin. Eleven independent studies investigated *BRCA1* hypermethylation [20, 29, 42, 48, 55, 56, 76, 78, 79, 93, 94]. Statistically significant results, correlating hypermethylation of this gene with poor disease outcome, were found in four studies, both in DNA from surgical resections and in blood serum. *PITX2* was studied in five independent studies, though it should be noted these studies were all performed by the same research group [40–42, 61, 69]. All but one study found a statistically significant association between *PITX2* hypermethylation and poor outcome; this correlation seemed to be location specific and was found in primary surgical resections and blood serum but not in serum derived from the bone marrow [40]. Five studies reported on RARB hypermethylation in relation to breast cancer recurrence [50, 56, 67, 79, 88]. A statistically significant correlation was found in two studies and proved to be independent of studied tissue type. *PGR* was studied in five independent cohorts in four different studies [64, 66, 73, 79]. Two alternative promotor sites were analysed in these studies; for both alternative promotors, significant correlation to poor survival was found. Three studies reported on *CDH1* methylation in relation to disease outcome [49, 50, 58]. Two studies identified a statistically significant correlation between *CDH1* hypermethylation and poor disease outcome. *PCDH10* was studied by two independent studies; both found *PCDH10* hypermethylation significantly correlated with poor prognosis [32, 59]. A combined analysis of *GSTP1*, *RASSF1* and *RARB* hypermethylation was the only gene panel analysed in two independent study populations [38, 39]. Fujita et al. studied this panel in blood serum derived from two independent study populations and found a strong correlation with poor overall survival in both cohorts.

For two methylation markers, significant correlations with both poor and improved disease outcome were reported. *GSTP1* was analysed in four studies [27, 54, 78, 79]. Three studies found promoter methylation of this gene to be associated with poor survival in multivariable analysis. However, Klajic et al. found *GSTP1* methylation to be strongly associated with better breast cancer-specific survival [54]. All four studies studied *GSTP1* promoter methylation in primary tumour resections; Sharma et al. also investigated the relation between *GSTP1* methylation detected in blood serum of breast cancer patients with the recurrence-free interval, but found no statistically significant correlation [78]. *ESR1* was studied in eight independent cohorts; in six cohorts, no statistically significant correlation with disease outcome was found [56, 62, 66, 68, 75, 79, 91]. Widschwendter et al. analysed the impact of ESR1 in two patient cohorts in a univariate analysis, one consisting of patients treated with tamoxifen and one consisting of patients that had not received tamoxifen. In the non-treated cohort, no statistically significant correlation was found; however, in the tamoxifen-treated cohort, a borderline statistical significance with improved disease-free survival was shown (HR 0.7; 95% CI 0.5–1.0) [91]. Ramos et al. found a strong correlation of *ESR1* hypermethylation and metastasis-free survival in a cohort of 67 patients; in this study, no details on treatment of the cohort were reported [75].

The markers *CCND2*, *SFRP2*, *PTPRO*, *CDNK2A* and *LINE-1* showed all a correlation for methylation and patient outcome in one study, but these effects were not validated in other studies reporting on these markers [42, 44, 45, 48, 50, 54, 56, 57, 63, 65, 72, 79, 83, 87].

Hypermethylation of the markers *SFN*, *APC*, *ID4* and *CDH13* was analysed in two independent sub-populations, but did not show any statistically significant correlation with disease outcome [48, 63, 66, 68, 70, 88, 92, 95].

## Discussion

In this systematic review, we provide an overview of prognostic DNA methylation markers for ER- and/or PR-positive breast cancer. We identified promoter hypermethylation of *RASSF1*, *BRCA1*, *PITX2*, *CDH1*, *RARB*, *PCDH10* and *PGR* as well as the marker panel *GSTP1*, *RASSF1* and *RARB* as possible markers of poor disease outcome. Four of these markers (*RASSF1*, *PITX2*, *PCDH10* and the panel) were also shown to be of prognostic value independently of clinically relevant prognostic factors, suggesting that these markers may provide additional prognostic information. This may help to identify patients at increased risk of disease recurrence and to inform the choice of adjuvant therapy.

Although promising, current LOE for these markers is low, either level 3 or 4. Several explanations can be suggested for this low LOE. Most studies were performed retrospectively, which provides a lower LOE as compared to prospectively designed studies. To overcome this, biomarker research should preferably select patients from previously established prospective cohorts [96]. In addition, only 18 markers and one marker panel were tested in multiple patient populations, and studies that did investigate the same marker showed extensive heterogeneity in technical assays, study endpoints and patient selection. This heterogeneity impaired comparison between studies and the performance of meta-analyses, making it impossible to combine low LOE studies in order to reach a higher LOE.

Heterogeneity between individual studies was introduced by several factors. DNA methylation can be analysed using several different techniques. Studies included in this systematic review applied nine different assays for determining methylation status. Although it has been shown that varying techniques could lead to different results [97, 98], this is not always the case. In previous research, we have shown that the prognostic impact of a DNA methylation biomarker is not affected by the applied technique if the chosen technique is optimised correctly [99]. Optimisation depends on correctly chosen cut-off values, assay conditions, origin and quality of the used source DNA and the location in which methylation is analysed [99–101]. These factors all determine whether a sample is identified as methylated or unmethylated, directly influencing the sensitivity and specificity of the assay and should therefore be reported in great detail [24, 25]. In our review, almost none of the included studies sufficiently reported these factors, as is also illustrated by a median REMARK score of 12. Recent research has shown the 5-hydroxy-methylation is a separate entity in epigenetic DNA alterations; however, as most currently applied techniques are incapable of discerning DNA methylation from 5-hydroxy-methylation, we have considered this distinction outside the scope of this review.

Apart from the chosen assay characteristics, heterogeneity in study endpoints was seen for the included studies. Although 85% of all studies reported the used endpoint, these endpoints were frequently not clearly described. Due to the long median survival in early breast cancer patients, overall survival is generally not feasible as an endpoint. Therefore, surrogate endpoints relating to disease recurrence are often applied. Recurrence in breast cancer can have many forms, such as locoregional recurrence, distant recurrence or second primary disease. As different types of recurrence are related to different patient, tumour and treatment characteristics, a precise definition of surrogate endpoints is needed [23]. In addition, endpoint selection should be tailored to the envisioned purpose of the envisioned marker. For example, when a marker is studied with the goal of predicting the risk distant recurrence, distant recurrence-free survival or distant recurrence-free interval would include the most relevant events [23].

Differences in tumour and treatment characteristics between studies were an additional source of heterogeneity. The treatment patients received, the percentage of patients that had hormone receptor-positive breast cancer or amplification of the HER2 gene differed markedly. Moreover, these characteristics, though vital for interpretation of the results of the studies, were often reported incompletely. The treatment regimen was only specified in 65% of the included studies. When treatment was specified, it was often described as ‘according to local guidelines’, which can vary per region, but also per time period. In breast cancer, the status and prognostic effect of biomarkers may change due to a specific treatment and it should therefore be considered when interpreting study results [102]. The risk of breast cancer recurrence is directly correlated to the ER, PR and HER2 status [5–7]. A lack of a detailed description of the study population makes it difficult to perform a meta-analysis or to identify a clinical setting in which a marker may be of use [24, 25]. In addition, there was also a great variation in the covariates used in the multivariable analyses. In order to interpret the prognostic value of a marker, at least all currently used clinical prognostic factors, i.e. TNM classification, tumour grade, ER status, PR status and HER2 status, should be included [24, 25]. Many studies did not perform these analyses or omitted key covariates without explanation.

The studies summarised in this review show numerous promising DNA methylation biomarkers for hormone receptor-positive breast cancer. Unfortunately, a meta-analysis of these studies is not possible due to the differences between the included studies. Additional research is needed to establish the prognostic value of these markers in predicting distant recurrence when used in addition to existing tests. Future research should be designed to prevent selection and confounding bias and should report findings in adherence to the REMARK criteria. In addition, measurement bias should be prevented by the usage of internationally accepted endpoints reported in the STEEP guidelines for breast cancer endpoint reporting [23]. In order to get closer to clinical implementation, studies with a higher LOE are warranted. A feasible strategy may be to select patients from previously established prospective cohorts [96].

In this review, we have not addressed the rational mechanistic pathways linking the investigated markers to breast cancer recurrence, as in many of the included studies this aspect is not explored. Functional exploration of epigenetic markers can help in marker validation as it adds a hint towards causation, which often lacks in observational epigenetic research [18]. However, if a marker is thoroughly validated, it can be of clinical use without being mechanistically understood [18]. We acknowledge that the REMARK criteria were designed as reporting guidelines and not as a tool for quality assessment. As reporting quality and study quality are not synonymous, the REMARK score is not a quality indicator as such, although we did find a relation between the REMARK score and reporting of statistically significant results. The REMARK score should not be regarded as a rating, but as a tool used to identify weaknesses in research. Some included studies analysed methylation as a side objective, rather than a main study objective, resulting in less well-described methodology and thus poor REMARK performance. A low REMARK score should therefore not be mistaken for an indicator of a poor marker, but rather an indication this marker needs further investigation.

## Conclusion

In this systematic review, we provided a comprehensive overview of the available literature on prognostic DNA methylation biomarkers in ER- and/or PR-positive breast cancer. We identified hypermethylation of *RASSF1*, *BRCA1*, *PITX2*, *CDH1*, *RARB*, *PGR*, *PCDH10* and a panel of *GSTP1*, *RASSF1* and *RARB* as potential markers of poor disease outcome. We also provided an analysis of study reporting, which indicates high heterogeneity in currently published literature on this subject. Future prognostic DNA methylation marker research would benefit from standardised DNA methylation assessment methods, thorough study reporting and the use of standardised endpoint definitions.

## Supplementary information

**Additional file 1: Tables S1.** Complete search terms pubmed and embase. Complete overview of keywords and equivalents used for the literature search in pubmed and embase.

**Additional file 2: Table S2.** REMARK checklist for scoring the reviewed studies as published by McShane et al. British journal of cancer. 2005;93 (4):387–91. REMARK checklist used for calculating the remark score for all included studies.

**Additional file 3: Table S3.** studies, studied markers and outcomes. Review Data Table. Complete review data containing all included studies, markers and outcome values.

**Additional file 4: Table S4.** Risk of potential bias and confounders of the included studies. Table indicating risks for potential bias for all included studies.

**Additional file 5: Table S5.** Results table of multiple investigated markers. Table summarising all results from markers that were investigated in two or more independent study populations.

**Additional file 6: FigureS6.** Correlation of single tested markers with prognosis. Overview of all markers tested in a single study population and reported correlation with prognosis. *Italic* markers do not correspond to Ref-Seq registered genes.

**Additional file 7: Table S7.** Correlation of methylation marker panels with prognosis. Correlation of methylation marker panels with prognosis in early stage breast cancer. Overview of all marker panels tested in a single study population and reported correlation with prognosis.

## Data Availability

Not applicable.

## Abbreviations

CI: Confidence interval
CpG: Cytosine guanine dinucleotide
ER: Oestrogen receptor
HR: Hazard ratio
LOE: Level of evidence
PR: Progesterone receptor
PRISMA: Preferred Reporting Items for Systematic Reviews and meta-Analyses
REMARK: REporting recommendations for tumour MARKer prognostic studies
STEEP: The proposed Standardized definitions for Efficacy EndPoints in adjuvant breast cancer trials
