# Supplementary material for: Prognostic DNA methylation markers for hormone receptor breast cancer: a systematic review
Source: Breast Cancer Res. 2020 Jan 31;22:13. doi: 10.1186/s13058-020-1250-9 (PMC6993426; doi:10.1186/s13058-020-1250-9)
Supplement: Supplementary file 1 — Additional file 1: Tables S1. Complete search terms pubmed and embase. Complete overview of keywords and equivalents used for the literature search in pubmed and embase. [file 13058_2020_1250_MOESM1_ESM.docx]

| **Free term** | **Mesh term** |
| --- | --- |
| Breast neoplasm  Breast neoplasms | Breast neoplasm  Breast neoplasms |
| Breast cancer | Breast cancer |
| Methylation marker | No mesh term |
| Dna methylation  Dna methylations  Methylation, dna  Methylations,dna | Dna methylation  Dna methylations  Methylation, dna  Methylations,dna |
| Luminal A  Luminal B  luminal | luminal |
|  |  |
| Estrogen receptor, Estrogen receptors  Oestrogen receptor, oestrogen receptors | Estrogen receptors |
| hormone positive | No mesh term |
| Progesterone receptor  Progesterone receptors | Progesterone receptor  Progesterone receptors |
| ER positive | No mesh term |
| PR positive | No mesh term |
|  |  |
| Therapy prediction | No mesh term |
| Prognoses  Prognosis | Prognoses  prognosis |
| Prognostic marker | No mesh term |
| Predictive marker | No mesh term |
| Survival  Analysis, survival  Analyses, survival  Overall survival | Analysis, survival  Analyses, survival |
| Analysis, Kaplan meier kaplan meier analysis  Curves, Kaplan meier survival  Survival curves, Kaplan meier  kaplan meier survival curves  Kaplan meier survival | Analysis, Kaplan meier kaplan meier analysis  Curves, Kaplan meier survival  Survival curves, Kaplan meier  kaplan meier survival curves |
| Disease-free survival  DFS  Disease free survival  Disease free survivals | Disease free survival  Disease free survivals |
| Recurrence  recurrences | Recurrence  Recurrences |
|  |  |
| **IDFS**  **Invasive disease-free survival**  **Invasive disease free survival** | No mesh term |
| **DDFS**  **Distant disease-free survival**  **Distant disease free survival** | No mesh term |
| **DRFS**  Distant relapse-free survival  Distant relapse free survival | No mesh term |
| **RFS  recurrence-free survival**  **Recurrence free survival** | No mesh term |

| **Recurrence-free interval**  **Recurrence free interval** | No mesh term |
| --- | --- |
| **Breast cancer-free interval**  **Breast cancer free interval** | No mesh term |
| **Distant recurrence-free interval**  **Distant recurrence free interval** | No mesh term |

Search terms pubmed

**(((((breast neoplasm[MeSH Terms]) OR breast neoplasms[MeSH Terms]) OR breast neoplasm) OR breast neoplasms) OR breast cancer[MeSH Terms]) OR breast cancer**

**(((((((((((((((luminal[MeSH Terms]) OR luminal A) OR luminal B) OR luminal) OR estrogen receptors[MeSH Terms]) OR estrogen receptors) OR estrogen receptor) OR oestrogen receptor) OR oestrogen receptors) OR Progesterone receptors) OR hormone positive) OR progesterone receptor[MeSH Terms]) OR progesterone receptors[MeSH Terms]) OR Progesterone receptor) OR ER positive) OR PR positive**

**((((((((methylation marker) OR dna methylation[MeSH Terms]) OR dna methylation) OR dna methylations[MeSH Terms]) OR dna methylations) OR methylation, dna) OR methylation, dna[MeSH Terms]) OR methylations, dna[MeSH Terms]) OR methylations, dna**

**(((((((((((((((((((((((((((((((((((((((((((((((((((therapy prediction) OR prognoses) OR prognosis) OR prognosis[MeSH Terms]) OR prognoses[MeSH Terms]) OR prognostic marker) OR predictive marker) OR survival) OR analysis, survival) OR analyses, survival) OR overall survival) OR analysis, survival[MeSH Terms]) OR analyses, survival[MeSH Terms]) OR analysis, kaplan meier) OR kaplan meier analysis) OR curves, kaplan meier survival) OR survival curves, kaplan meier) OR kaplan meier survival curves) OR kaplan meier survival) OR analysis, kaplan meier[MeSH Terms]) OR kaplan meier, analysis[MeSH Terms]) OR curves, kaplan meier survival[MeSH Terms]) OR survival curves, kaplan meier[MeSH Terms]) OR kaplan meier survival curves[MeSH Terms]) OR disease-free survival) OR DFS) OR disease free survival) OR disease free survivals) OR disease free survival[MeSH Terms]) OR disease free survivals[MeSH Terms]) OR recurrence[MeSH Terms]) OR recurrences[MeSH Terms]) OR recurrence) OR recurrences) OR IDFS) OR Invasive disease-free survival) OR Invasive disease free survival) OR DDFS) OR Distant disease-free survival) OR Distant disease free survival) OR DRFS) OR Distant relapse-free survival) OR Distant relapse free survival) OR RFS) OR recurrence free survival) OR recurrence-free survival) OR Recurrence-free interval) OR Recurrence free interval) OR Breast cancer free interval) OR Breast cancer-free interval) OR Distant recurrence-free interval) OR Distant recurrence free interval**

Search embase:

1) Breast neoplasm OR Breast neoplasms OR breast cancer

2) methylation marker OR Dna methylation OR Dna methylations OR methylation, dna OR methylations, dna

3) luminal A OR luminal B OR luminal OR estrogen receptor OR estrogen receptors OR oestrogen receptor OR oestrogen receptors OR hormone positive OR progesterone receptor OR progesterone receptors OR ER positive OR PR positive

4) Therapy prediction OR prognoses OR prognosis OR prognostic marker OR predictive marker OR survival OR analysis, survival OR overall survival OR OS OR analysis, kaplan meier OR kaplan meier analysis OR Curves, kaplan meier survival OR survival curves, kaplan meier OR kaplan meier survival curves OR kaplan meier survival OR disease-free survival OR DFS OR disease free survival OR disease free survivals OR recurrence OR recurrences OR IDFS OR Invasive disease-free survival OR invasive disease free survival OR DDFS OR Distant disease-free survival OR distant disease free survival OR DRFS OR distant relapse-free survival OR distant relapse free survival OR RFS OR recurrence-free survival OR recurrence free survival OR recurrence-free interval OR recurrence free interval OR breast cancer-free interval OR breast cancer free interval OR distant recurrence-free interval OR distant recurrence free interval

result 23.2.16: 313 results
