# Supplementary material for: Prognostic DNA methylation markers for hormone receptor breast cancer: a systematic review
Source: Breast Cancer Res. 2020 Jan 31;22:13. doi: 10.1186/s13058-020-1250-9 (PMC6993426; doi:10.1186/s13058-020-1250-9)
Supplement: Supplementary file 2 — Additional file 2: Table S2. REMARK checklist for scoring the reviewed studies as published by McShane et al. British journal of cancer. 2005;93 (4):387–91. REMARK checklist used for calculating the remark score for all included studies. [file 13058_2020_1250_MOESM2_ESM.docx]

Table S2. REMARK checklist for scoring the reviewed studies as published by McShane *et al.* British journal of cancer. 2005;93(4):387-91.

| REMARK checklist item | Description |  |
| --- | --- | --- |
| Introduction |  |  |
| 1 | State the marker examined, study objectives and pre-specified hypotheses. |  |
| Materials and Methods |  |  |
| Patients |  |  |
| 2 | Describe the characteristics (eg disease stage or co-morbidities) of study patients, including their source and inclusion and exclusion criteria |  |
| 3 | Describe treatments received and how chosen (eg randomized or rule-based). |  |
| Specimen characteristics |  |  |
| 4 | Describe the type of biological material used (incl. control samples) and methods for preservation. |  |
| Assay methods |  |  |
| 5 | Specify the assay method used and provide (or reference) a detailed protocol, incl. specific reagents or kits used, quality control procedures, reproducibility assessment, quantitation methods, and scoring and reporting protocols. Specify whether and how assays were performed blinded to the study endpoint. |  |
| Study design |  |  |
| 6 | State the method of case selection, including whether prospective or retrospective and whether stratification or matching (eg by stage of disease or age) was used. Specify the time period from which cases were taken, the end of the follow-up period, and the median-follow-up time. |  |
| 7 | Precisely define all clinical endpoints examined. |  |
| 8 | List all candidate variables initially examined or considered for inclusion in models. |  |
| 9 | Give rational for sample size; if the study was designed to detect a specified effect size, give the target power and effect size. |  |
| Statistical analysis methods |  |  |
| 10 | Specify all statistical methods, including details of any variable selection procedures and other model-building issues, how model assumptions were verified, and how missing data were handled. |  |
| 11 | Clarify how marker values were handled in the analyses; if relevant, describe methods used for cutpoint determination. |  |
| Results |  |  |
| Data |  |  |
| 12 | Describe the flow of patients through the study, including the number of patients included in each stage of the analysis (a diagram may be helpful) and reasons for dropout. Specifically, both overall and for each subgroup extensively examined report the number of patients and the number of events. |  |
| 13 | Report distributions of basic demographic characteristics (at least age and sex), standard (disease-specific) prognostic variables, and tumor marker, including number of missing values. |  |
| Analysis and interpretation |  |  |
| 14 | Show the relation of the marker to standard prognostic variables |  |
| 15 | Present univariable analysis showing the relation between the marker and outcome, with the estimated effect (eg hazard ratio and survival probability). Preferably provide similar analyses for all other variables being analyzed. For the effect of a tumor marker on a time-to-event outcome, a Kaplan-Meier plot is recommended. |  |
| 16 | For key multivariable analyses, report estimated effects (eg hazard ratio) with confidence intervals for the marker and, at least for the final model, all other variables in the model. |  |
| 17 | Among reported results, provide estimated effects with confidence intervals from an analysis in which the marker and standard prognostic variables are included, regardless of their statistical significance. |  |
| 18 | If done, report results of further investigations, such as checking assumptions, sensitivity analysis, and internal validation. |  |
| Discussion |  |  |
| 19 | Interpret the results in the context of the pre-specified hypotheses and other relevant studies; include a discussion of limitations of the study. |  |
| 20 | Discuss implications for further research and clinical value. |  |
