# Supplementary material for: Prognostic DNA methylation markers for hormone receptor breast cancer: a systematic review
Source: Breast Cancer Res. 2020 Jan 31;22:13. doi: 10.1186/s13058-020-1250-9 (PMC6993426; doi:10.1186/s13058-020-1250-9)
Supplement: Supplementary file 4 — Additional file 4: Table S4. Risk of potential bias and confounders of the included studies. Table indicating risks for potential bias for all included studies. [file 13058_2020_1250_MOESM4_ESM.docx]

Supplemental Table S4 Risk of potential bias and confounders of the included studies.

Studies indicated by a “X“ potentially have an increased risk of bias, whereas studies indicated by a “√“ potentially have a decreased risk of bias.

|  | Selection bias | Assay method  (measurement bias) | Outcome assessment (measurement bias) | Confounders |  |
| --- | --- | --- | --- | --- | --- |
| **Study** |  |  |  |  |  |
| Jahangiri,2018 | **√** | **X** | **X** | **√** |  |
| Müller, 2003 | **X** | **√** | **√** | **X** |  |
| Liu, 2018 | **X** | **√** | **X** | **√** |  |
| Buhmeida, 2011 | **√** | **√** | **√** | **√** |  |
| Kioulafa, 2009 | **X** | **√** | **√** | **√** |  |
| Xu, 2013 | **X** | **√** | **√** | **√** |  |
| Mirza, 2012 | **X** | **√** | **√** | **X** |  |
| Arai, 2006 | **X** | **√** | **√** | **√** |  |
| McCormack, 2008 | **X** | **√** | **√** | **X** |  |
| Nimmrich, 2008 | **√** | **√** | **√** | **√** |  |
| Xu, 2009 | **√** | **√** | **√** | **√** |  |
| Gobel, 2011 | **X** | **X** | **√** | **√** |  |
| Lu, 2011 | **X** | **X** | **√** | **X** |  |
| Martins, 2011 | **X** | **√** | **√** | **√** |  |
| Cerne, 2012 | **√** | **√** | **√** | **X** |  |
| VanHoesel, 2012 | **√** | **√** | **√** | **√** |  |
| VanHoesel, 2012 (2) | **√** | **√** | **√** | **√** |  |
| Ulrisch, 2013 | **X** | **√** | **√** | **√** |  |
| Shen, 2015 | **X** | **X** | **X** | **X** |  |
| Palmieri, 2012 | **X** | **√** | **√** | **X** |  |
| Noetzel, 2008 | **X** | **√** | **√** | **X** |  |
| Dietrich, 2010 | **√** | **√** | **√** | **X** |  |
| Ramos, 2010 | **X** | **√** | **√** | **√** |  |
| Xu, 2012 | **X** | **√** | **√** | **√** |  |
| Klajic, 2013 | **X** | **√** | **√** | **√** |  |
| Martinez Galan, 2014 | **X** | **√** | **√** | **X** |  |
| Li, 2014 | **X** | **√** | **√** | **√** |  |
| Hill, 2011 | **X** | **√** | **X** | **X** |  |
| Rodriguez, 2008 | **X** | **√** | **√** | **X** |  |
| Kioulafa, 2009 | **X** | **√** | **√** | **X** |  |
| Mirza, 2010 | **X** | **√** | **√** | **X** |  |
| Iorns, 2008 | **X** | **√** | **√** | **X** |  |
| Mehrotra, 2004 | **X** | **√** | **√** | **X** |  |
| Fiegl, 2008 | **X** | **X** | **√** | **X** |  |
| Hartmann, 2009 | **√** | **√** | **√** | **√** |  |
| Veeck, 2008 | **X** | **√** | **√** | **√** |  |
| Li, 2006 | **X** | **√** | **√** | **X** |  |
| Pathiraja, 2011 | **√** | **√** | **√** | **X** |  |
| Weissenborn, 2017 | **√** | **√** | **√** | **X** |  |
| Sharma, 2009 | **X** | **√** | **√** | **√** |  |
| Widschwenter, 2004 | **X** | **√** | **√** | **√** |  |
| Fiegl, 2005 | **√** | **√** | **√** | **√** |  |
| Hill, 2010 | **X** | **√** | **X** | **X** |  |
| Chen, 2009 | **√** | **√** | **√** | **X** |  |
| Maier, 2007 | **√** | **√** | **X** | **X** |  |
| Farynja, 2012 | **X** | **√** | **√** | **X** |  |
| Fu, 2010 | **X** | **√** | **X** | **X** |  |
| Kioulafa, 2009 | **X** | **√** | **√** | **√** |  |
| Zhang, 2015 | **√** | **√** | **√** | **√** |  |
| Jung, 2013 | **√** | **√** | **X** | **X** |  |
| Harbeck, 2008 | **√** | **√** | **√** | **√** |  |
| Karray Chouayekh, 2010 | **X** | **√** | **X** | **X** |  |
| Huang, 2013 | **X** | **√** | **√** | **X** |  |
| Sharma, 2010 | **X** | **√** | **√** | **X** |  |
| Veeck, 2008 (2) | **X** | **√** | **√** | **X** |  |
| Chimonidou, 2013 | **X** | **√** | **√** | **X** |  |
| Ebeid, 2016 | **√** | **√** | **√** | **X** |  |
| Stefansson, 2015 | **X** | **√** | **√** | **X** |  |
| Angelova, 2012 | **√** | **√** | **√** | **X** |  |
| Krasteva, 2012 | **X** | **√** | **√** | **√** |  |
| Fujita, 2014 | **X** | **√** | **√** | **X** |  |
| Liu, 2016 | **X** | **√** | **√** | **X** |  |
| Jing, 2008 | **X** | **√** | **√** | **X** |  |
| Fujita, 2012 | **√** | **√** | **√** | **√** |  |
| Wee, 2012 | **X** | **X** | **X** | **X** |  |
| Stephen, 2016 | **X** | **√** | **√** | **X** |  |
| Veeck, 2008 | **X** | **√** | **√** | **X** |  |
| Perez Janices, 2015 | **X** | **√** | **√** | **X** |  |
| Park, 2014 | **X** | **√** | **X** | **X** |  |
| Wang, 2012 | **X** | **√** | **X** | **X** |  |
| Fang, 2011 | **X** | **√** | **√** | **X** |  |
| Ren, 2013 | **X** | **√** | **√** | **√** |  |
|  |  |  |  |  |  |
