# Supplementary figures and images for: Prognostic DNA methylation markers for hormone receptor breast cancer: a systematic review
Source: Breast Cancer Res. 2020 Jan 31;22:13. doi: 10.1186/s13058-020-1250-9 (PMC6993426; doi:10.1186/s13058-020-1250-9)

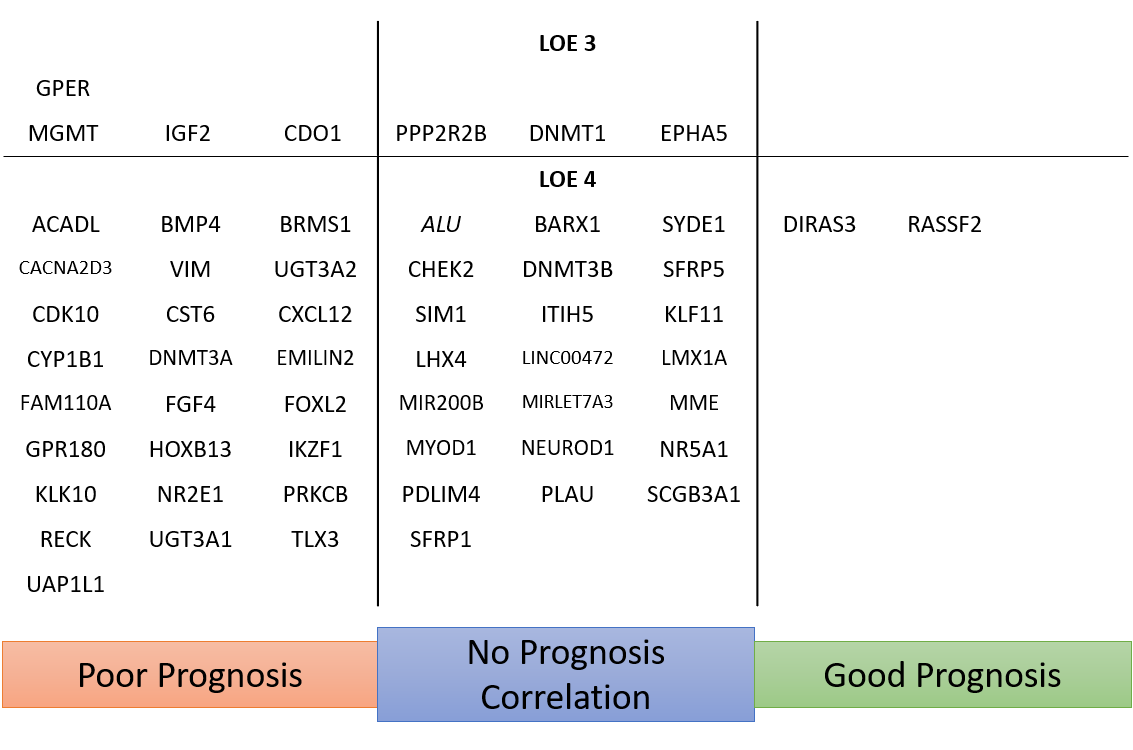

Supplement: Supplementary file 6 — Additional file 6: FigureS6. Correlation of single tested markers with prognosis. Overview of all markers tested in a single study population and reported correlation with prognosis. Italic markers do not correspond to Ref-Seq registered genes. [file 13058_2020_1250_MOESM6_ESM.tif]
