# Supplementary material for: Prognostic DNA methylation markers for hormone receptor breast cancer: a systematic review
Source: Breast Cancer Res. 2020 Jan 31;22:13. doi: 10.1186/s13058-020-1250-9 (PMC6993426; doi:10.1186/s13058-020-1250-9)
Supplement: Supplementary file 7 — Additional file 7: Table S7. Correlation of methylation marker panels with prognosis. Correlation of methylation marker panels with prognosis in early stage breast cancer. Overview of all marker panels tested in a single study population and reported correlation with prognosis. [file 13058_2020_1250_MOESM7_ESM.docx]

**Table S7. Correlation of methylation marker panels with prognosis in early stage breast cancer**

|  | Markers |
| --- | --- |
| Correlated with poor prognosis | PITX2+RASSF1, PITX2+BMP4+FAM110A+FGF4, BRCA1+CDKN2A, ESR+RARB, RASSF1+APC, PGR+ESR1+RASSF1+CDKN2A+BRCA1+GSTP1+RARB, TTBK1+ZNF132+KCNA2, MINT17+MINT31+RARB, |
| Correlated with good prognosis | ALX4+ARHGEF7+RASGRF2, RASSF1+RARB+CDH1+CDKN2A, |
| Not correlated with prognosis | RASSF1+PCDH10, RASSF1+RARB+CDH13 |
